# TFF1 in Aqueous Humor—A Potential New Biomarker for Retinoblastoma

**DOI:** 10.3390/cancers14030677

**Published:** 2022-01-28

**Authors:** Maike Anna Busch, André Haase, Natalia Miroschnikov, Annika Doege, Eva Biewald, Nikolaos E. Bechrakis, Manfred Beier, Deniz Kanber, Dietmar Lohmann, Klaus Metz, Nicole Dünker

**Affiliations:** 1 Center for Translational Neuro- and Behavioral Sciences, Institute of Anatomy II, Department of Neuroanatomy, Medical Faculty, University of Duisburg-Essen, 45147 Essen, Germany; andre.haase@uk-essen.de (A.H.); Natalia.miroschnikov@uk-essen.de (N.M.); annika.doege@uk-essen.de (A.D.); nicole.duenker@uk-essen.de (N.D.); 2 Department of Ophthalmology, Medical Faculty, University of Duisburg-Essen, 45147 Essen, Germany; eva.biewald@uk-essen.de (E.B.); nikolaos.bechrakis@uk-essen.de (N.E.B.); 3 Institute of Human Genetics, Medical Faculty, Heinrich-Heine University, 40225 Düsseldorf, Germany; beierm@uni-duesseldorf.de; 4 Institute of Human Genetics, Medical Faculty, University of Duisburg-Essen, 45147 Essen, Germany; deniz.kanber@uk-essen.de (D.K.); Dietmar.lohmann@uk-essen.de (D.L.); 5 Institute of Pathology, Medical Faculty, University of Duisburg-Essen, 45147 Essen, Germany; Klaus.metz@uk-essen.de

**Keywords:** retinoblastoma, RB, trefoil factor family peptides, TFF1, aqueous humor, liquid biopsy, biomarker

## Abstract

**Simple Summary:**

Retinoblastoma is the most common pediatric intraocular malignancy with high cure rates in developed countries. Nevertheless, useful predictive biomarkers providing reliable evidence for therapy decisions are urgently needed to optimize therapy regimes. TFF1 is a promising candidate as it is expressed in a more advanced subtype of retinoblastoma. Additionally, TFF1 is a naturally secreted peptide. Thus, TFF1 might be detectable in the aqueous humor of RB patients’ eyes, providing the opportunity to determine its expression prior to therapy without the necessity of a tumor biopsy. We therefore investigated for the first time aqueous humor samples of retinoblastoma patients in order to test for the availably and expression status of TFF1 as well as to compare it with the original tumor and established corresponding primary cell cultures.

**Abstract:**

Retinoblastoma (RB) is the most common childhood eye cancer. The expression of trefoil factor family peptide 1 (TFF1), a small secreted peptide, has been correlated with more advanced RB stages and it might be a promising new candidate as a RB biomarker. The study presented addressed the question of if TFF1 is detectable in aqueous humor (AH) of RB patients’ eyes, providing easy accessibility as a diagnostic and/or therapy accompanying predictive biomarker. The TFF1 expression status of 15 retinoblastoma AH samples was investigated by ELISA and Western blot analyses. The results were correlated with the TFF1 expression status in the tumor of origin and compared to TFF1 expression in established corresponding primary tumor cell cultures and supernatants. Nine out of fifteen AH patient samples exhibited TFF1 expression, which correlated well with TFF1 levels of the original tumor. TFF1 expression in most of the corresponding primary cell cultures reflects the levels of the original tumor, although not all TFF1-expressing tumor cells seem to secret into the AH. Together, our findings strongly suggest TFF1 as a reliable new RB biomarker.

## 1. Introduction

Retinoblastoma (RB) is the most common pediatric intraocular malignancy with an incidence rate of about 1 in 17,000 live births [1,2]. Although some patients develop metastases, survival rates are over 90% in high-income countries, whereas in low-income countries, tumors remain undiagnosed and grow to an advanced, globe-threatening stage [3]. Therefore, the main therapeutic focus lies on life-saving treatment regimens including ocular tumor treatment and prevention of metastatic spread [4]. A second critical goal of RB therapy is maximization of eye and visual preservation [4]. For a long period, biopsies or the removal of eye fluid were contraindicative due to the risk of tumor dissemination [5]. However, today, paracentesis of aqueous humor (AH) is a standard procedure of the protocol for intravitreal chemotherapy injections [6] and the risk of induced extraocular spread is considered extremely low [7]. This circumstance offers the possibility for a completely new diagnostic and prognostic RB procedure using AH of patients for the evaluation of biomarkers prior to or during therapy [8]. Over the last years, different studies investigated AH in enucleated RB eyes and unraveled several markers supposed to either provide clinical values for diagnosis and clinicopathological associations or reflecting response to treatment regimens or putative therapy targets ( for review see: [8]). 

Our group identified trefoil factor family peptide 1 (TFF1) as a putative new RB marker correlating with higher clinical tumor-node-metastasis (TNM) stage and poorly differentiated tumor cells [9]. In addition, we could show that TFF1 functionally acts as tumor suppressor gene in RB cell lines [10] and is epigenetically regulated [11]. In a most recent study by Liu et al., the relevance of TFF1 as potential marker for RB was confirmed and connected to a subtype of RBs associated with a higher risk of metastasis [4]. TFF peptides are ectopically expressed in different human tumors (for review see: [12]) and three members—TFF1, TFF2, and TFF3—of this family of small secreted proteins have been characterized in mammals so far. TFFs possess a characteristic clover leaf-like disulfide structure, the so-called TFF domain. They are highly expressed in the gastrointestinal tract with main functions in the maintenance and protection of epithelial surfaces [13,14,15]. Additionally, TFFs are expressed in the central nervous system, in ocular tissues, and in the murine retina (for review see: [12,16]). Previous studies by our group revealed that TFF1 is not expressed in the healthy human retina, whereby RB cell lines and RB tumors exhibit variable levels of TFF1 [9,11,17]. Thus, TFF1 is a promising candidate as a predictive biomarker in aqueous humor of RB patients, as it is a naturally secreted peptide, which is highly expressed in RB patients harboring a higher risk of metastasis. In the study presented, we investigated TFF1 expression in AH via ELISA and Western blot and correlated the expression patterns with clinical parameters and TFF1 expression in the original tumor tissue of the enucleated patients’ eyes. Additionally, we compared the TFF1 status of the corresponding RB primary cell culture cells with TFF1 levels in the tumor of origin. We showed that TFF1 is indeed detectable in the AH of patients eyes increasing its availability as a potential biomarker. 

## 2. Materials and Methods

### 2.1. Human Retinoblastoma and Retina Samples

Human retinoblastoma (RB) primary tumor material, aqueous humor samples, and paraffin sections from enucleations of 15 patients, as well as post-mortem retina samples from cornea donors were used for comparative TFF1 expression studies. The Ethics Committee of the Medical Faculty of the University of Duisburg-Essen approved the use of human retina (approval # 06-30214) and retinoblastoma samples (approval # 14-5836-BO) for research conducted in the course of the study presented and written informed consent was obtained from patients’ relatives or parents.

Primary tumor material and aqueous humor samples were harvested immediately after enucleation. Aqueous humor was aspirated via an anterior chamber puncture using a 30 G needle. In the next step, the actual tumor was removed from the globe via scleral fenestration. Aqueous humor samples were stored at −80 °C until use and the tumor tissue samples were cultured as described subsequently. 

This study includes a case series of 15 untreated eyes from individual children diagnosed with intraocular retinoblastoma between 2020 and 2021. Diagnosis was confirmed pathologically after enucleation. All RB samples were reviewed by a specialized pathologist. The data collected included patient’s age at diagnosis, gender, laterality, RB1 mutation status, tumor volume, optic nerve, and choroid invasion. 

### 2.2. Cell Lines and Primary RB Cell Culture

The human RB cell lines Rbl13 and Rbl30 were kindly provided by Dr. H. Stephan. The cell lines were cultivated as suspension cultures in Dulbecco’s modified Eagle’s medium (DMEM; PAN-Biotech, Aidenbach, Germany) with 15% fetal calf serum (FCS; PAN-Biotech, Aidenbach, Germany), 100 U penicillin/mL and 100 µg streptomycin/mL (Invitrogen, Darmstadt, Germany), 4 mM L-glutamine (Sigma-Aldrich, München, Germany), 50 µM ß-mercaptoethanol (Carl-Roth, Karlsruhe, Germany), and 10 µg insulin/mL (Sigma-Aldrich, München, Germany) at 37 °C, 10% CO_2_, and 95% humidity as described previously (Busch et al. 2015). 

The primary RB tumor material was initially cut into small pieces with a sterile scalpel and subsequently washed three times in PBS with a centrifugation step in between (800 rpm for 2 min). After the last washing step, the tumor material was cultivated under the cell culture conditions described above. Cell culture supernatants were harvested and residual cells were removed by centrifugation. Cell culture supernatants were kept at −20 °C until usage.

### 2.3. TFF1 ELISA Analysis

The aqueous humor samples of RB patients and the supernatant of the corresponding primary cell cultures as well as the supernatant of two RB cell lines (Rbl-13 and Rbl-30) were analyzed with a human TFF1 ELISA kit (ab213833, abcam, Cambridge, UK) following the manufacturer´s protocol. Fifty microliters sample volume was used and the concentration of the analyzed sample concentrations (pg) was calculated based on the standard curve included in the kit. 

### 2.4. Immunocytochemistry and Immunfluorescence Stainings

For the cohort of 15 retinoblastomas included in this study, automated immunostaining for CRX and Ki-67 was performed on 5 µm paraffin-embedded samples with the OptiView DAB IHC detection kit (Santa Cruz Biotechnology, Dallas, TX, USA) or UltraView alkaline phosphatase red detection kit (Ventana Roche, Grenzach-Wyhlen, Germany). TFF1 immunostaining was performed with the Vectastin Elite ABC kit (Vector Laboratories, Burlingame, CA, USA) as previously described by our group [9]. The following antibodies were used: anti-CRX (1:50, Santa Cruz Biotechnology, Dallas, TX, USA, clone A-9), anti-Ki-67 (ready-to-use, Ventana Roche, Grenzach-Wyhlen, Germany, clone 30-9), and TFF1 (1:200, abcam, Cambridge, UK, # ab92377). Images were acquired using an Aperio ScanScope AT2 (Leica, Wetzlar, Germany) slide scanner. Two researchers, blind for additional patient information, independently assessed each stained slide by eyeballing, taking into account different staining intensities (I) defined as null (0), mild (1), moderate (2), or strong (3), and the percentage (P) of tumor cells with CRX stained nuclei and TFF1 positive cytoplasm. The quick score (QS) was then calculated as I × P (from 0 to 300). The TFF1 intensities 1 and 2 were combined and specified as “expressed”, whereas intensity 0 was specified as “not expressed” and intensity 3 as “highly expressed”.

For immunofluorescence staining, 1 × 10^5^ cells were seeded on poly-D-lysine (Sigma, Hamburg, Germany) coated coverslips. Cells were fixed with 4% paraformaldehyde (PFA; Sigma-Aldrich, St. Louis, MO, USA) for 1 h at 4 °C washed three times with phosphate buffered saline (PBS; pH 7.4) and permeabilized with 100% methanol for 10 min at room temperature. Cells were washed with PBS and blocked with PBS containing 0.3% Triton™ X-100 (Sigma), 4% bovine serum albumin (BSA; Carl-Roth, Karlsruhe, Germany), and 5% normal goat serum (NGS; Dako, Glostrup, Denmark) for 1 h at room temperature. The primary antibody used (incubated overnight at 4 °C) was TFF1 1:200 (abcam, Cambridge, UK, # ab92377) diluted in PBS with 0.3% Triton™ X-100, 4% BSA, and 5% NGS. A goat anti-rabbit antibody labeled with Alexa Flour^®^594 (Invitrogen, Darmstadt, Germany), diluted 1:1000 in PBS with 0.3% Triton™ X-100, 4% BSA, and 5% NGS was used to visualize the reaction. In order to stain the nuclei, cells were counter-stained with 4’,6-Diamidino-2-phenylindole (DAPI; Sigma, Hamburg, Germany). As controls, PBS was substituted for the primary antisera in order to test for non-specific labeling. Pictures were taken with a NIKON Eclipse E600 microscope equipped with a digital camera and NIKON Eclipse net software.

### 2.5. Western Blotting

Equal volumes of AH samples and cell culture supernatants were separated on a 12% SDS-PAGE and transferred onto nitrocellulose membranes. The primary antibodies used (incubated overnight at 4 °C) were TFF1 (1:1000 abcam, Cambridge, UK, # ab92377) and ß-actin (1:1000): #4967, Cell Signalling Technology (Cambridge, UK). The secondary antibody used was HRP-conjugated goat-anti-rabbit antibody (1:10,000); P0448; Dako (Glostrup, Denmark). Signals were developed by the WesternBright Chemiluminescence Reagent (Advansta, San Jose, CA, USA).

### 2.6. Statistical Analysis

Statistical analysis of tumor clinical parameters was correlated with the TFF1 expression in aqueous humor identified by ELISA analysis. Statistical analyses (Kruskal–Wallis rank sum *p*-values) were performed in the R statistical environment, version 3.2.0.16. Statistical analyses of the real-time PCR data were performed using GraphPad Prism 6. Results were analyzed by a Student’s *t*-test and considered significantly different if * *p* < 0.05, ** *p* < 0.01, or *** *p* < 0.001.

## 3. Results

### 3.1. Soluble TFF1 Is Detectable in Aqueous Humor of RB Patients and Primary Cell Culture-Derived Supernatant

**Figure 1:**
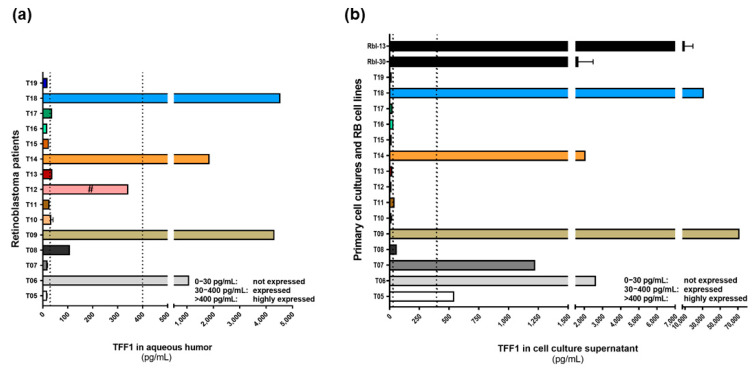
TFF1 expression analyses in aqueous humor samples of RB patients and corresponding primary cell culture supernatants. (**a**) TFF1 ELISA analysis of 15 aqueous humor samples of RB patients, showing four patients with highly expressed TFF1 (>400 pg/mL), five patients with expressed TFF1 (30–400 pg/mL), and six patients without TFF1 expression (0–30 pg/mL) in the aqueous humor. (**b**) The supernatants of corresponding primary cell cultures of RB patient tumors shown in (**a**) revealed six highly TFF1 expressing samples, three samples expressing TFF1, and six samples without TFF1 expression. The supernatant of two RB cell lines (Rbl-13 and Rbl-30) highly expressing and secreting TFF1 are used as internal positive controls. Vertical dotted lines indicate three TFF1 expression levels; #: sample was a vitreous body aspirate.

TFF1 is expressed in a subpopulation of RB tumors and seems to be correlated with more advanced tumor stages. Therefore, soluble TFF1 is potentially secreted from the tumor cells into the aqueous humor of RB patients. In order to investigate the accessibility of soluble TFF1 in aqueous humor, we tested AH samples of 15 RB patients by TFF1 ELISA (Figure 1a). We could show that RB tumor cells indeed secrete measurable amounts of soluble TFF1 into the aqueous humor of RB patients’ eyes. We defined three TFF1 expression groups: group I) not expressed (0–30 pg/mL), group II) expressed (30–400 pg/mL), and group III) highly expressed (>400 pg/mL), based on the ELISA analysis. We identified four tumors secreting very high TFF1 concentrations (between 1000 and 4500 pg/mL; T06, T09, T14, and T18) into the AH, five tumors secreting moderate levels of TFF1 (T08, T10, T12, T13, and T17), and six tumors without TFF1 secretion into the AH (T05, T07, T11, T15, T16, and T19). Subsequently, we investigated if corresponding primary cell culture cells established from the original patient tumor samples also secrete TFF1 into the cell culture supernatant. Two RB cell lines with a known TFF1 secretion potential were used as positive controls. We could show that cultured primary RB cells from patients with TFF1 positive AH samples secrete TFF1 into the supernatant (Figure 1b), except for the cell culture of specimen T12. Two RB cell cultures (T05 and T07) do secrete TFF1 into the supernatant, whereas the corresponding AH samples were negative for TFF1. Thus, we could show that TFF1 is detectable in the AH of RB patients and that corresponding primary cell cultures mimic patients’ TFF1 status, indicating their suitability as an in vitro RB model system. 

**Figure 2:**
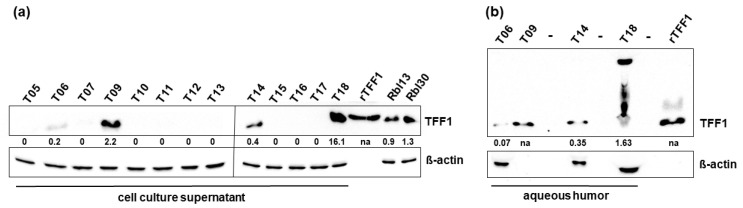
Western blot analysis of TFF1 expression in cell culture supernatants of primary cell cultures and aqueous humor samples of RB patients. (**a**) Western blot analysis of primary cell culture supernatants showing TFF1 expression in the highly TFF1 expressing tumors of the ELISA analysis (T06, T09, T14, and T18). Recombinant TFF1 (rTTF1) and the RB cells lines Rbl13 and Rbl30 served as positive controls. (**b**) TFF1 expression levels revealed by Western blot analysis for the four AH samples with high TFF1 expression levels in the ELISA assay. The indicated intensity ratios relative to ß-actin, used as a loading control, were calculated using MICRO MANAGER 1.4 software (University of California, San Francisco, CA, USA). -: empty lanes; na: not analyzed.

In order to verify the TFF1 ELISA results, cell culture supernatants were analyzed by Western blot (Figure 2a and Figure S1) and compared to the TFF1 levels of supernatant from the RB cell lines Rbl13 and Rbl30 serving as positive controls. TFF1 expression was detectable by Western blot in samples with TFF1 status initially defined as “highly expressed” (<400 pg/mL TFF1) in ELISA analyses (T06, T09, T14, and T18), whereas all other samples analyzed were below the detection limit of this method. Analysis of AH of the highly TFF1 expressing sample group by Western blot confirmed the TFF1 expression pattern (Figure 2b and Figure S2). 

### 3.2. TFF1 Expression in Primary Cell Culture Cells

**Figure 3:**
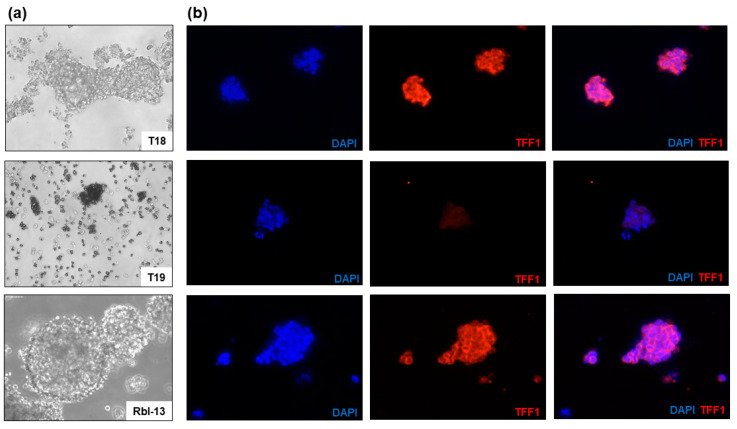
Immunofluorescence TFF1 staining of two primary RB cell cultures (T18 and T19) and RB cell line Rbl-13. (**a**) Morphology of the primary RB cell cultures T18 and T19 as well as RB cell line Rbl-13 revealed by phase contrast imaging (200×). (**b**) DAPI (**blue**), TFF1 (**red**), and merged DAPI/TFF1 immunofluorescence staining of the respective RB cells (200×). T18 and the positive control Rbl-13 showed a high expression of TFF1 in contrast to T19 cells without TFF1 expression.

In order to analyze the cellular expression of TFF1 in the established primary cultures immunofluorescence staining was performed. The intracellular TFF1 expression pattern correlates well with the TFF1 secretion status described above. We found primary RB cell cultures highly expressing TFF1 (Figure 3a,b, T18) as well as cell cultures without detectable TFF1 expression (Figure 3a,b, T19) in comparison to the RB cell line Rbl-13 used as positive control (Figure 3a,b). Real-time PCR analysis (Figure S3) revealed that in the samples analyzed, the intracellular *TFF1* mRNA expression does not correlate with the TFF1 protein concentrations measured in the AH and supernatant. Hence, *TFF1* mRNA levels are not reliable to predict expression, but TFF1 status needs to be evaluated on the protein level. 

### 3.3. TFF1 Expression in Primary RB Tumors

**Figure 4:**
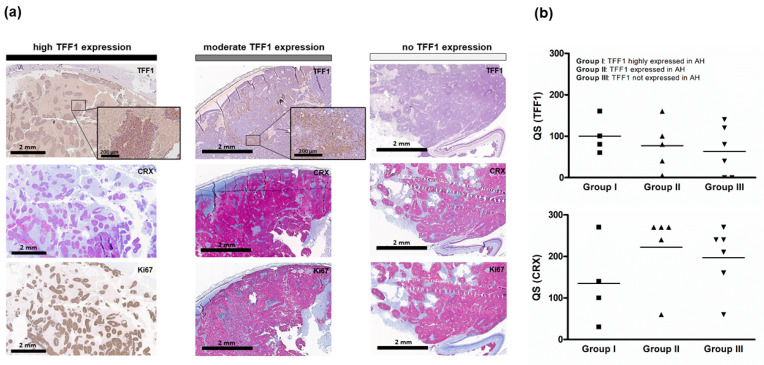
Histological analysis of primary RB tumors. (**a**) TFF1, CRX, and Ki67 expression levels in exemplary human RB tumor paraffin sections. Immunohistochemistry was revealed using diaminobenzidine detection (**brown signal**) or alkaline phosphatase detection (**red**) and hematoxylin counterstaining (**blue nuclei staining**). Scale bars, 2 mm and 200 µM (zoom box). (**b**) Dot plots showing the quick score (QS) for TFF1 and CRX in 15 tumors analyzed. One way ANOVA was used to assess the difference of the QS for group I (TFF1 highly expressed in AH) vs. group II (TFF1 expressed in AH) vs. group III (TFF1 not expressed in AH). No significant difference was detectable between the groups. The squares and triangles in (**b**) represent the different tumors per group.

To compare the expression of TFF1 in AH and supernatant of primary cultured RB cells with its expression pattern in original tumor specimens, paraffin sections of all tumors were immunocytochemically stained for TFF1. TFF1 immunostaining revealed highly TFF1 expressing tumors, tumors with moderate TFF1 expression, and tumors without TFF1 expression (Figure 4a). The corresponding CRX staining confirmed the RB nature of the tumors and Ki67 staining the proliferation activity of the tumor cells. All RB tumor sections stained positively for Ki67 indicating that the tumor cells were still proliferative. We showed that all tumors with detectable TFF1 in the AH samples analyzed stained highly or moderately positive for TFF1 in primary tumor sections. However, not all TFF1-positive RB tumors seemed to secrete TFF1 into the AH of the patients’ eye as quick score (QS) of TFF1 revealed a median QS of 60 for group III, which does not express TFF1 in AH (Figure 4b). No significant difference in TFF1 and CRX QS was detectable between the groups. 

Thus, TFF1 expression in AH samples correlates to 100% with a positive TFF1 expression pattern in the primary RB tumor, however, not all TFF1 expressing tumors necessarily secrete TFF1 into the AH of the patients.

### 3.4. Correlation of Clinical and Pathological Characteristics of the Analyzed RB Tumors

Table 1 summarizes the clinical and pathological characteristics of the 15 RB patients analyzed. In order to correlate these parameters with TFF1 expression in AH, we divided the patients into two groups: a TFF1 expressing (60%) group and a non-expressing group (40%). No statistically significant differences between both groups in relation to sex, *RB1* germline mutations, laterality, age at diagnosis, tumor volume, optic nerve invasion, or choroid invasion could be detected (Table 1). 

**Figure 5:**
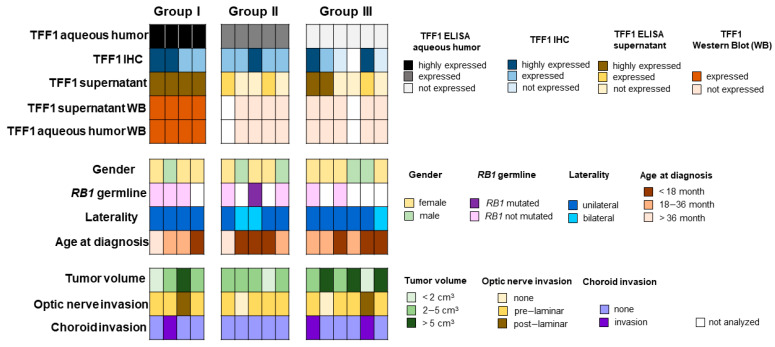
TFF1 expression and clinical characteristics of retinoblastoma samples. Classification of TFF1 expression in AH, RB tumors, and primary cell culture in relation to key clinical and pathological characteristics.

To combine and summarize all data, RB patients were categorized into three groups: group I “highly expressing TFF1”, group II “expressing TFF1”, and group III “without TFF1 expression” (Figure 5). In group I tumors, highly expressing TFF1 in AH (1) TFF1 could also be detected via Western Blot, (2) the original tumor stains highly positive for TFF1, and (3) primary cultured cells express and secrete TFF1. In group II tumors, TFF1 expression in the original tumor specimens correlates with the TFF1 expression in the AH. However, not all primary cell cultures secrete TFF1 into the supernatant. In group III, without detectable TFF1 in AH samples, TFF1 probably is not secreted into the AH even if the tumor bulk contained TFF1-expressing cells detectable by immunohistochemistry. TFF1 expression of group II and III is below the detection limit of Western blotting analysis. Taken together, this shows that TFF1 is indeed secreted into the AH of the patients’ eyes and is readily detectable via ELISA. Even if not all TFF1 positive RB tumors secrete TFF1 into the AH, no false positive TFF1 expression was measured in AH samples.

## 4. Discussion

Aqueous humor analyses and the identification of tumor biomarkers in eyes without enucleation have the potential to renew the management of retinoblastoma. For many years, any opportunity to obtain fluid-like AH from RB eyes was contraindicated in order to prevent tumor seeding [5,18,19]. However, today, AH paracentesis is part of the protocol for intravitreal chemotherapy injections and the risk of extraocular spread is considered extremely low [7,20,21,22,23,24]. Aqueous fluid is routinely aspirated prior to intravitreal injection to prevent reflux from the injection site. This standardized clinical procedure offers the possibility to readily access AH in RB eyes prior and during therapy and to evaluate biomarkers that may correlate with features of the tumor and provide diagnostic and prognostic value. Aqueous humor has been shown to give important information for intraocular diseases, including RB; however, previous studies used AH from enucleated eyes [8]. This is going to change in the near future due to the clinical applicability of AH sample aspiration and its availability for diagnosis, prognosis, and/or management of RB. 

Therefore, in the study presented, we intended to investigate the potential of TFF1 as a RB biomarker and its availability in AH of RB patients’ eyes. TFF1 is already described as a functional biomarker in several other tumor entities, e.g., breast cancer [25,26], esophageal squamous cell carcinoma [27], and gastric cancer [28]. In breast cancer, a correlation of high TFF1 expression in blood samples of patients with metastatic disease compared to those without metastatic disease has been demonstrated [29]. Evaluating TFF1 staining of tumor sections after enucleation, we and others already described TFF1 as a biomarker for a subset of RBs. Here, we analyzed AH, tumor of origin, and established corresponding primary cell culture cells of 15 RB patients for TFF1 expression and secretion status and compared the results with the clinical parameters. We showed that TFF1 is expressed in the AH of most patients analyzed. All patients with TFF1-positive AH also expressed TFF1 in the original tumor, whereas some RB tumors express TFF1 without secreting it into the AH. We would like to emphasize the fact that there was no false positive result within our AH analysis. It seems, however, that not all TFF1-positive RB tumors can be identified by AH sampling, probably due to a lower secreting rate of some TFF1-positive RB tumors or fewer TFF1-positive cells within the tumor bulk resulting in TFF1 concentrations in AH samples that are below the detection limit. This hypothesis is supported by the fact that those three RB tumors of group III (without measurable TFF1 in AH) that do express TFF1 protein in the original tumor also secrete TFF1 in measurable amounts into the supernatant of the corresponding primary cell cultures. Possibly, primary RB cells expressing TFF1 have a growth advantage in culture that leads to a higher concentration of TFF1 in the cell culture supernatant in comparison to the investigated AH samples of the original tumor specimens. Fortunately, the primary cultured RB tumor cells mimic the original RB tumor with regard to TFF1 expression and secretion status for most analyzed RB tumors, rendering them an excellent in vitro system for further TFF1-based RB studies. 

Correlation of the clinical and pathological characteristics of the investigated RB tumors with the TFF1 expression status in the AH of the patients’ eye did not allow for any stratification. The lack of correlation is most likely attributable to the comparatively small sample size, because we and others have already shown that TFF1 expression in RB tumors indeed correlates with clinical parameters [4,9]. Our group demonstrated that TFF1 correlates with a higher clinical tumor-node-metastasis (TNM) stage and poorly differentiated tumor cells [8] and a recent study showed that TFF1 is linked to RBs which are associated with a higher risk of metastasis, referred to as subtype 2 [4]. Summarizing, one can state that the identification of TFF1 in the AH of RB patients opens the field for new diagnostic approaches. Beside other AH markers already described as potentially useful for diagnosis or reflecting response to RB treatment regimens [30,31,32,33],TFF1 is a new potential valuable biomarker for subtype 2 RBs [4]. Additionally, we could show that ELISA is a reliable method for TFF1 diagnostics in AH as it is very sensitive (sensitivity < 10 pg/mL), the sample volume needed is below the volume routinely aspirated prior to chemotherapy and the assay time is short (3.5 h). Thus, we could show for the first time that TFF1 has potential as a clinically useful biomarker for future RB diagnostics. 

## 5. Conclusions

Aqueous humor analyses and identification of tumor biomarkers have the potential to renew advanced retinoblastoma management and to assure RB diagnosis in cases of clinically uncertain differential diagnosis. TFF1, a secreted peptide, is ectopically expressed in a subset of more advanced RB tumors and its expression correlates with a higher risk for metastases. We provided evidence for TFF1 expression in the AH of RB patients, strongly suggesting TFF1 as a clinically interesting new RB biomarker. 

## Figures and Tables

**Table 1:** Clinical and pathological characteristics of RB patients stratified by TFF1 expression in aqueous humor. NA: not available, *n*: number in each group, *N*: total number. * Kruskal–Wallis rank sum *p*-value.

	TFF1 Expressed *n* (%)	TFF1 Not Expressed *n* (%)	*N*	*p-*Value *
**Patients**	9 (60)	6 (40)	15	
**Sex**				1
Female	6 (67)	4 (67)	10	
Male	3 (33)	2 (33)	5	
***RB1*** **germline mutations**				0.88
Yes	1 (11)	0 (0)	1	
No	5 (56)	2 (33)	7	
NA	3 (33)	4 (67)	7	
**Laterality**				0.79
Unilateral	7 (78)	5 (83)	12	
Bilateral	2 (22)	1 (17)	3	
**Age at diagnosis**				0.47
<18 month	4 (45)	3 (50)	7	
18–36 month	3 (33)	3 (50)	6	
>36 month	2 (22)	0 (0)	2	
**Tumor volume**				0.26
<2 cm^3^	2 (22)	1 (17)	3	
2–5 cm^3^	6 (67)	2 (33)	8	
>5 cm^3^	1 (11)	3 (50)	4	
**Optic nerve invasion**				0.89
None	1 (11)	1 (17)	2	
Pre-laminar	7 (78)	4 (67)	11	
Post-laminar	1 (11)	1 (17)	2	
**Choroid invasion**				0.30
None	8 (89)	4 (67)	12	
Invasion	1 (11)	2 (33)	3	
**Pre enucleation MRI**				0.76
Choroid invasion	2 (22)	2 (33)	4	
Optic nerve invasion	3 (33)	3 (50)	6	

## Data Availability

The data presented in this study are available on request from the corresponding author.

## Supplementary Materials

The following are available online at https://www.mdpi.com/article/10.3390/cancers14030677/s1, Figures S1 and S2: Full Western blot images. Figure S3: TFF1 mRNA expression of primary RB tumor cells and RB cell lines analyzed by real-Time PCR.
