# Supplementary material for: TFF1 in Aqueous Humor—A Potential New Biomarker for Retinoblastoma
Source: Cancers (Basel). 2022 Jan 28;14(3):677. doi: 10.3390/cancers14030677 (PMC8833755; doi:10.3390/cancers14030677)
Supplement: Supplementary file 1 [file cancers-14-00677-s001.zip › cancers-1564714-supplementary.pdf]

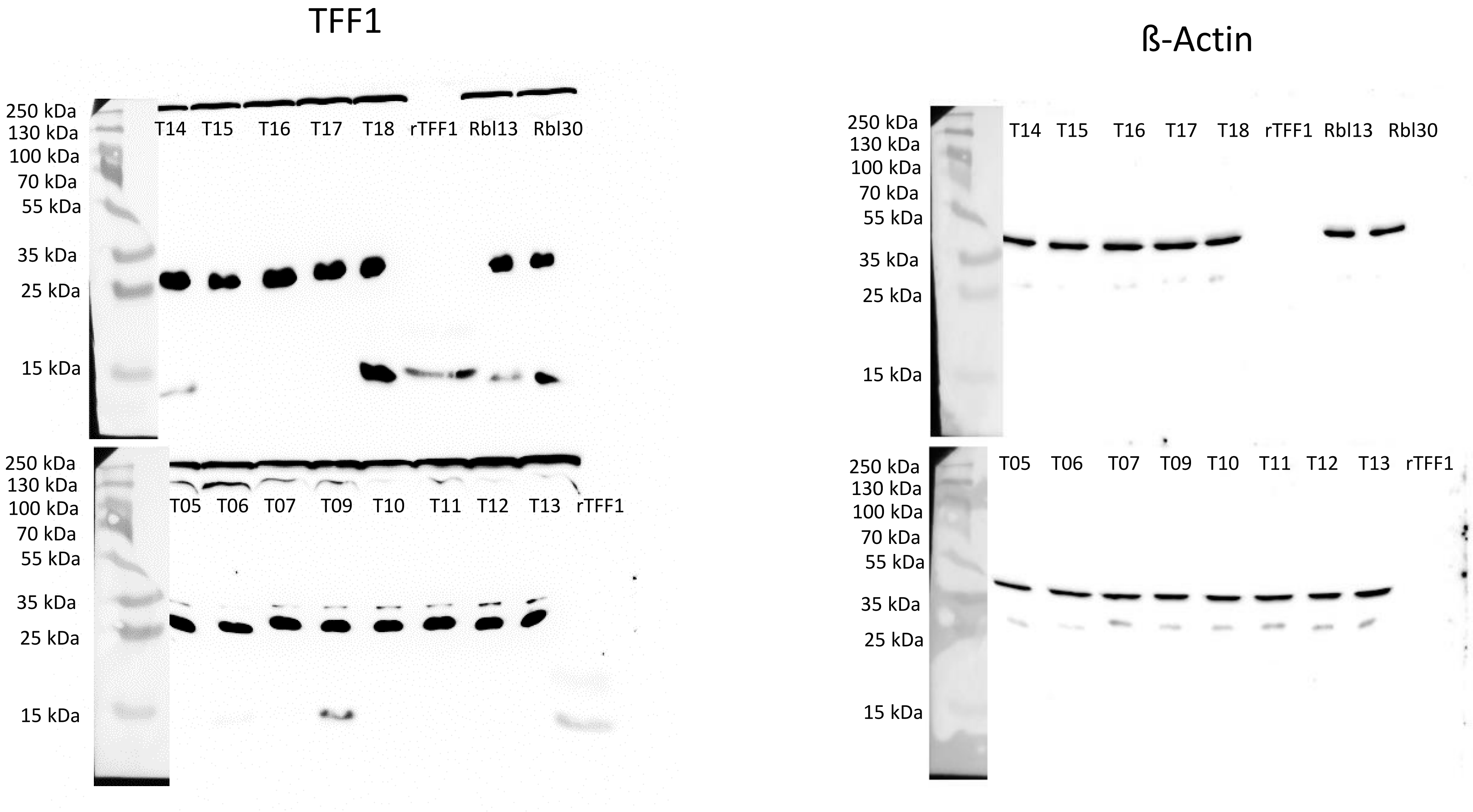

Figure S1. Full Western Blots of Figure 2a.

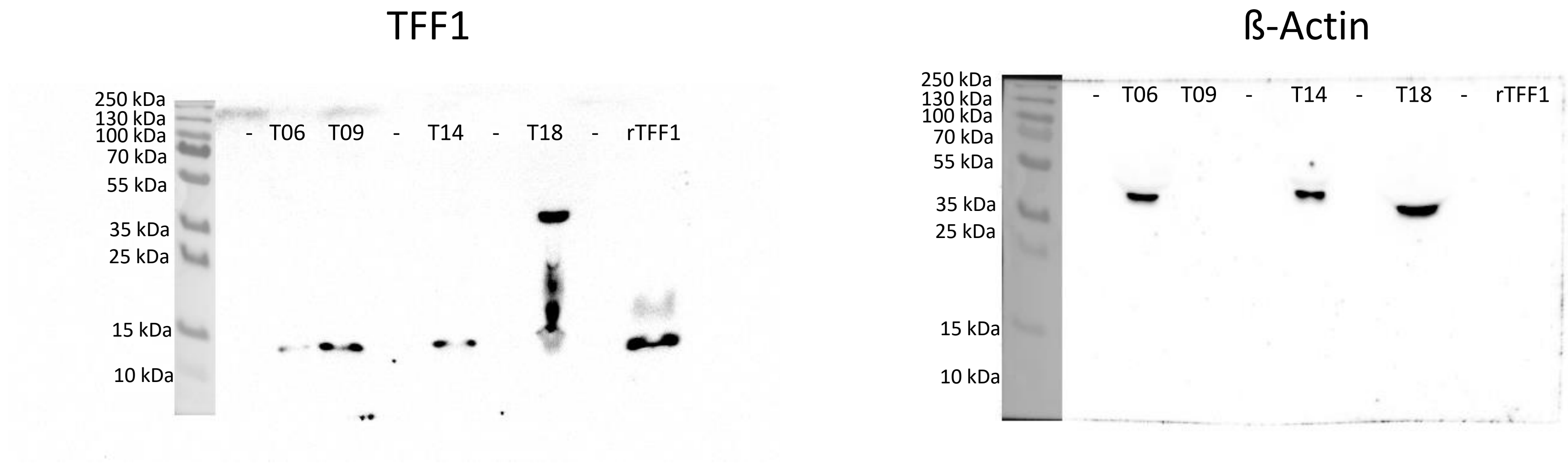

Figure S2. Full Western Blots of Figure 2b.

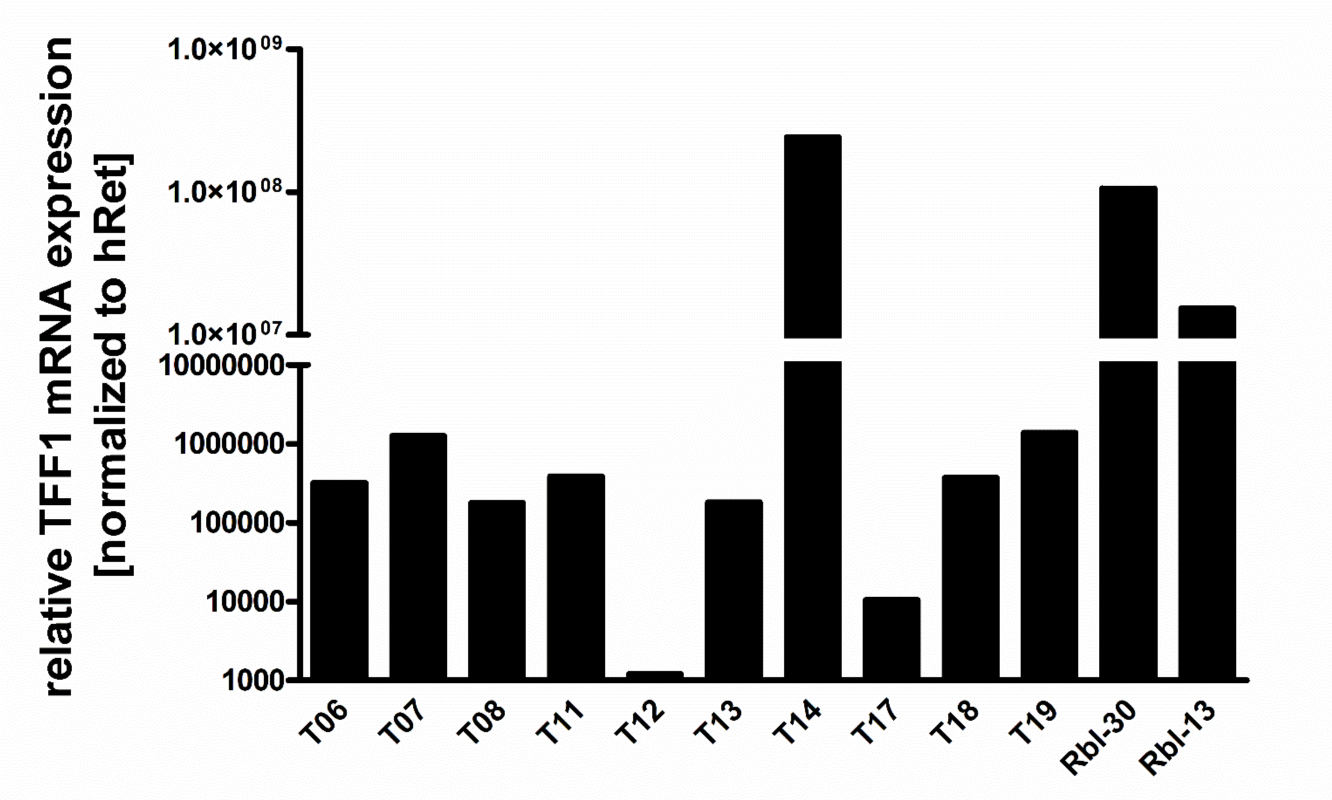

Figure S3. TFF1 mRNA expression of primary RB tumor cells and RB cell lines analyzed by Real-Time PCR.
